# Computation of diffuse scattering arising from one-phonon excitations in a neutron time-of-flight single-crystal Laue diffraction experiment

**DOI:** 10.1107/S1600576715010912

**Published:** 2015-07-08

**Authors:** Matthias J. Gutmann, Gabriella Graziano, Sanghamitra Mukhopadhyay, Keith Refson, Martin von Zimmerman

**Affiliations:** a ISIS Facility, Rutherford Appleton Laboratory, Chilton, Didcot, Oxfordshire OX11 0QX, UK; b Thomas Young Centre, London Centre for Nanotechnology and Department of Chemistry, University College London, London WC1E 6BT, UK; c Department of Physics, Royal Holloway, University of London, Egham TW20 0EX, UK; d Deutsches Elektronen Synchrotron DESY, Notkestrasse 85, Hamburg 22603, Germany

**Keywords:** neutron time-of-flight, Laue diffraction, single crystals, phonon excitation

## Abstract

Direct phonon excitation in a neutron time-of-flight single-crystal Laue diffraction experiment has been observed in a single crystal of NaCl. At room temperature both phonon emission and excitation leave characteristic features in the diffuse scattering and these are well reproduced using *ab* *initio* phonons from density functional theory (DFT). A measurement at 20 K illustrates the effect of thermal population of the phonons, leaving the features corresponding to phonon excitation and strongly suppressing the phonon annihilation. A recipe is given to compute these effects combining DFT results with the geometry of the neutron experiment.

## Abstract 2

A methodology is presented to compute diffuse scattering arising from one-phonon excitations in a time-of-flight neutron single-crystal Laue diffraction experiment from density functional theory results. This methodology is illustrated using NaCl as an example.

## 1. Introduction   

The most common type of diffuse scattering at room-temperature in a single-crystal diffraction experiment designed to measure it is caused by lattice vibrations and is known as thermal diffuse scattering (TDS) (Born & Lonsdale, 1942 ▸). This can be seen using laboratory or synchrotron X-ray sources as well as neutrons at large-scale facilities (Osborn & Welberry, 1990 ▸; Welberry & Goossens, 2008 ▸). In principle, such data can be used to reconstruct phonon dispersion curves at least for simple crystal structures and using model potentials (Holt *et al.*, 1999 ▸). Diffuse scattering was among the early techniques used to obtain phonon dispersion curves along high-symmetry directions in elemental crystals before inelastic neutron and X-ray experiments became routine (Olmer, 1948 ▸; Placzek & Van Hove, 1954 ▸, Squires, 1978 ▸; Burkel, 2000 ▸). Inelastic techniques using single crystals provide complete momentum and energy-resolved dispersion curves, whilst diffraction only has momentum resolution. Interesting effects were noted by Willis *et al.* in the case of time-of-flight (TOF) Laue neutron diffraction where the neutron can accidentally match the wavevector and energy of phonons leading to a splitting of the thermal diffuse scattering features corresponding to phonon emission and absorption (Willis *et al.*, 1986 ▸; Schofield & Willis, 1987 ▸). This effect has not been observed with X-rays owing to the high energy of the order of several keV compared with up to hundreds of meV for lattice vibrations but in principle takes place as well. Willis derived a formalism to extract the sound velocity of a crystal using the splitting of the diffuse lines of the acoustic phonons which dominate the TDS pattern, at least close to the Bragg peaks (Willis, 1986 ▸). A few experiments were conducted to demonstrate the feasibility, and good agreement was obtained for the sound velocity obtained by other means (Willis, 1986 ▸; Carlile & Willis, 1989 ▸; Carlile *et al.*, 1992 ▸). Inelastic effects have also been noticed in quartz but were not considered further (Tucker *et al.*, 2001 ▸). It should be noted though that very few observations of direct phonon excitation in neutron diffraction experiments have been reported to date, limiting somewhat the use of this technique for molecular crystals in particular, with benzil being the only known example to the best of our knowledge (Welberry *et al.*, 2003 ▸).

Nowadays, density functional theory (DFT) has established itself as a tool of choice to predict phonon dispersion curves from a crystal structure and is routinely used in the planning and interpretation of inelastic experiments (Mitchell *et al.*, 2005 ▸). These computations also give the phonon eigenvectors for arbitrary *q* points which are not easily accessible experimentally though, in principle, they are available from the intensity of the TDS or inelastic peaks provided the inelastic structure factor differs from zero. Examples are now emerging where DFT computations are used to aid in the interpretation of diffuse scattering experiments (Bosak *et al.*, 2009 ▸; Wehinger *et al.*, 2014 ▸; Gutmann *et al.*, 2013 ▸).

In this paper, we combine DFT calculations with the geometry of the Laue neutron diffraction experiment to compute the ‘inelastic diffraction’ associated with phonons. This is compared with diffuse scattering calculated in the instrument geometry using the quasi-static approximation, which is when the phonon energy is negligible compared with the energy of the scattering probe. Our motivation is to be able to identify and reproduce the diffuse scattering arising from one-phonon scattering over a large region in reciprocal space in combination with *ab* *initio* calculations, in order to aid in the interpretation of diffuse scattering patterns beyond the Monte Carlo modelling approach (Welberry, 2004 ▸).

## 2. Experimental   

### 2.1. Diffraction experiments   

NaCl single crystals with a purity of 99.99% were purchased from Sigma–Aldrich; hereafter, they are referred to as synthetic. A large, irregularly shaped crystal was shaped into a sphere of 6 mm diameter. This sample is usually used as a calibration standard to ensure reproducibility between ISIS experiment cycles. Natural specimens were obtained in Hallstatt (Austria) from the local salt mine. They were of various colour, indicating impurities, but there were also colourless, optically clear crystals. An optically clear, colourless crystal of approximate dimensions 2 × 4 × 5 mm was extracted. However, the purity is not known explicitly and hence data from the synthetic crystal are used throughout the paper. The natural sample was only used to verify that effects similar to those described below occur and hence are sample independent, although they may vary in magnitude owing to impurity effects on the phonons (Caldwell, 1967 ▸). Neutron diffraction data were collected at room temperature using the SXD single-crystal diffractometer at the ISIS spallation neutron source (Oxfordshire, UK; Keen *et al.*, 2006 ▸) on various occasions on the synthetic crystal with exposures ranging between four hours and up to two days at single settings or a series of crystal settings about 20° apart. This crystal was also run at 20 K. The natural specimen was exposed for 12 h in various orientations but only at room temperature. Reciprocal-space volumes from these data were obtained using *SXD2001* (Gutmann, 2005 ▸). A natural crystal was also used for a complementary high-energy X-ray diffraction experiment on the beamline BW5 installed at the storage ring DORIS III at DESY in Hamburg, Germany. These data merely confirmed the absence of inelastic excitation effects seen in the neutron data.

### 2.2. Computation of phonon dispersions using DFT   

Electronic structure calculations were performed using the plane-wave pseudopotential methods as implemented in the *CASTEP* code (Clark *et al.*, 2005 ▸). The Perdew–Burke–Ernzerhof (Perdew *et al.*, 1996 ▸) generalized gradient approximation functional was used for both the plane-wave calculation and the generation of pseudopotentials. These were of the optimized norm-conserving variety (Rappe *et al.*, 1990 ▸). Energies and forces were well converged at a plane-wave cutoff of 600 eV. A Brillouin zone sampling of 8 × 8 × 8 *q* points (60 points when symmetry reduced) was found to be sufficient to converge energy and atomic forces below 2.2 × 10^−8^ eV per ion and 1.0 × 10^−3^ eV Å^−1^, respectively. Sufficient self-consistent cycles were performed to achieve a convergence tolerance of 1.0 × 10^−10^ eV per atom. Geometry optimizations used the Broyden–Fletcher–Goldfarb–Shanno algorithm with a force tolerance of 1.0 × 10^−3^ eV Å^−1^. Phonon dispersion calculations were performed on the resulting minimum-energy structures *via* diagonalization of dynamical matrices computed using density functional perturbation theory (DFPT) and linear-response methods (Refson *et al.*, 2006 ▸) on a total of 60 *q* points. Because our intention was to enable the program to use output from either *CASTEP* or *VASP/PHONOPY*, similar computations were also carried out with *VASP* (Kresse & Hafner, 1994 ▸), which equally well reproduced the diffraction features. Throughout the paper, the *CASTEP* calculations are used.

## 3. Computation of the thermal diffuse scattering   

**Figure 1:**
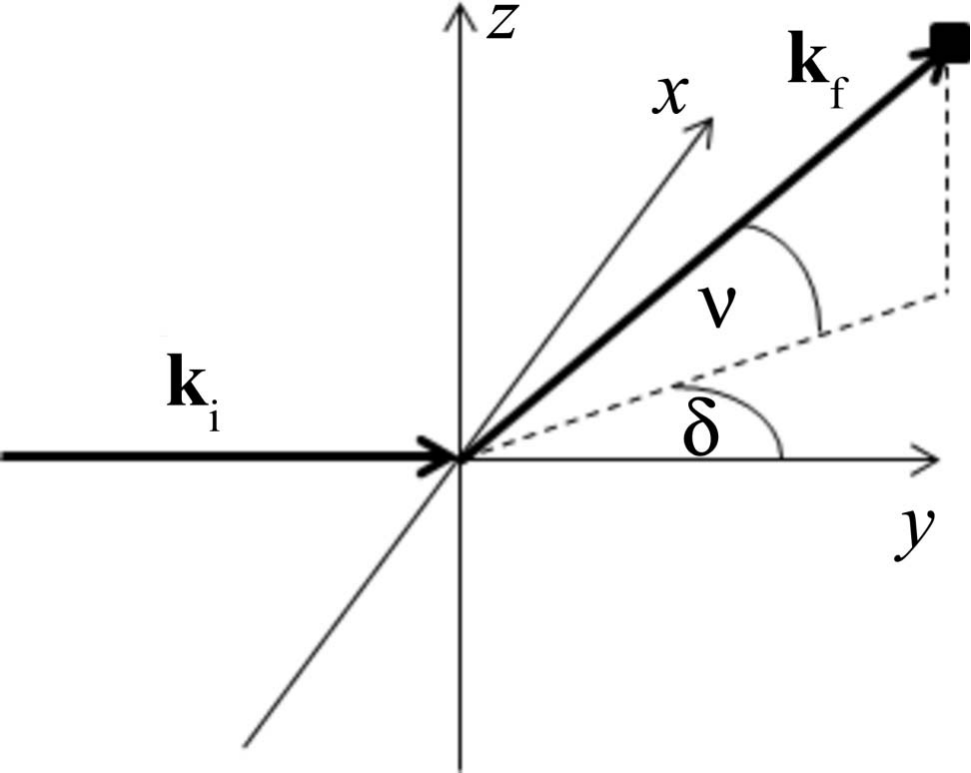
Coordinate system used to describe the scattering and instrument geometry.

**Figure 2:**
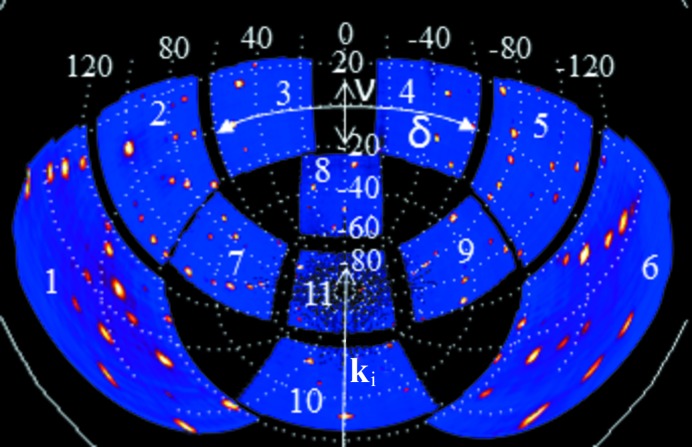
Illustration of the SXD detector geometry with detector module number labelling corresponding to Table 1 ▸ using an NaCl room-temperature data set integrated between a time-of-flight of 1500 and 10 000 microseconds. The coordinate system corresponding to Fig. 1 ▸ is superimposed as dotted grid lines.

We first establish a coordinate system in the neutron diffractometer based on spherical coordinates. The location of each pixel on the detector is characterized by two angles, δ and ν, which correspond to the longitude and latitude, respectively, as illustrated in Fig. 1 ▸. The incident neutron beam travels along 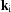, which is along the positive **y** direction, and is scattered into a pixel located at the end-point of 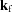. Expressed in the left-handed orthonormal coordinate system (*x*, *y*, *z*) shown in Fig. 1 ▸, the incident and scattered wavevectors read 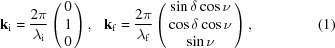
where λ_i_ and λ_f_ are the wavelengths (Å) of the incident and scattered neutrons, respectively. In the case of an elastic scattering event these wavelengths have the same magnitude, but they generally differ in the case of inelastic scattering. Table 1 ▸ lists the δ and ν angles for the 11 detector modules of the SXD instrument, and they are illustrated graphically in Fig. 2 ▸ for an NaCl data set at room temperature.

The angular ranges for the δ and ν angles in equation (1) for the various detectors of the SXD instrument are also given in Table 1 ▸. For a single crystal, the scattering vector 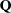 is 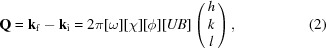
where [ω], [χ] and [φ] denote rotation matrices corresponding to the angle settings of the goniometer stage, [*UB*] is the orientation matrix of the crystal, and *h*, *k*, *l* are Miller indices, which need not be integer valued (Busing & Levy, 1967 ▸). For phonons it is convenient to decompose *h*, *k*, *l* into an integer part, where Bragg reflection may occur, and a fractional part such that the latter reflects the wavevector of the phonon in the first Brillouin zone.

In TOF neutron scattering, every pixel has a time-of-flight range associated with it, as a fixed histogram with a given number of time channels and a fixed minimum and maximum time. More recently event-mode has become available, where neutron events are time stamped as they occur without imposing a histogram. The SXD instrument at ISIS uses the fixed histogram-mode. The time-of-flight recorded corresponds to the total travel time of the neutron from the source to the detector pixel and is measured in units of microseconds. The total travel time 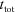 can be decomposed into the time from the source to the sample, *t*_i_, and the time from the sample to the pixel, *t*_f_. These components can be related to the wavelengths of the incident and scattered neutron:
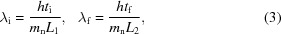
where *h* is the Planck constant, *m*_n_ is the neutron mass, *L*_1_ is the primary flight path (mm) from source to sample and *L*_2_ is the secondary flight path (mm) from the sample to the pixel. The energy (meV) of the neutron is related to the wavelength as follows:
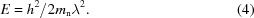
For calculating the TDS in the instrument geometry, every pixel and TOF channel is converted to *E*_f_ for a given *E*_i_ and the scattering vector 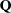 which is transformed back to *h*, *k*, *l* using equation (1) and decomposed into an integer and fractional *h*, *k*, *l* in the first Brillouin zone in order to look up the eigenvectors and phonon frequencies at that point in the DFT output. We note that the DFT calculations sometimes use a reduced cell compared with the conventional crystallographic cell and hence a transformation between these two cells should also be applied when converting to *h*, *k*, *l* of the DFT cell.

For calculating first-order TDS it is convenient to define a one-phonon structure factor (Xu & Chiang, 2005 ▸):
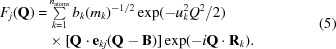
The summation extends over all *n*_atoms_ atoms in the unit cell. Further, *m*_*k*_ is the mass of atom *k*, *b*_*k*_ is its coherent scattering length, 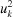 is the mean-square atomic displacement, taken as isotropic for the case of NaCl, 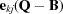 is the complex-valued eigenvector of atom *k* in the phonon mode *j* at wavevector 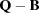 in the first Brillouin zone, with 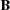 being the nearest Bragg point, and 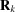 is the position of atom *k* in the unit cell. Here, unit cell refers to the primitive unit cell used by the *DFT* program. It should be noted that the form of equation (1) depends on whether the dynamical matrix in the *DFT* program is defined such as to give periodic eigenvectors, as is the case in *CASTEP* (Refson *et al.*, 2006 ▸), or otherwise, as is the case in *PHONOPY* (Togo *et al.*, 2008 ▸). The formula given here applies to *CASTEP*. For the case of nonperiodic eigenvectors see work by Xu & Chiang (2005 ▸).

The TDS intensity can be written as follows assuming energy and momentum conservation are fulfilled:
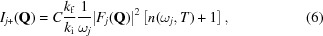


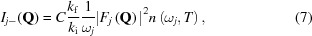
with *C* = *N*/2 and *N* being the number of atoms in the sample. This constant is treated as a scale factor and is set to 1 for convenience. ω_*j*_ denotes the frequency of the phonon mode *j*, and ‘+’ and ‘−’ refer to phonon creation and annihilation, respectively. The energy integration of the double-differential cross section [equations (6) and (7)] is effectively carried out here numerically using many wavelengths and counting the number of phonons falling into a given *Q* bin and normalizing to this. The phonon occupancy at temperature *T* is given by
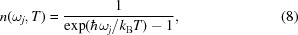
where ℏ is the reduced Planck constant and *k*_B_ is the Boltzmann constant.

In the quasi-static approximation, (6) and (7) are summed over all *n*_modes_ phonon modes to yield
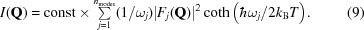
The constant is treated again as a scale factor and set to one. Initially a master mapping from pixel coordinates and TOF to a reciprocal-space map is established, assuming only elastic events. This master mapping is essentially assigning a given pixel and TOF channel to a voxel in the reciprocal-space volume. The recipe to compute inelastic diffraction can be summarized as follows:

(1) Select a fixed wavelength λ_i_ and a fixed energy E_i_, and 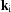.

(2) Compute for all pixels and TOF of the instrument λ_f_, E_f_ and 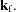


(3) Compute 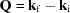 as well as 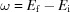 and use the experimental *UB* matrix for a given experimental run to derive *h*, *k*, *l*.

(4) Transform *h*, *k*, *l* from the conventional crystallographic cell to the DFT cell, if necessary, and decompose it into an integer part and a fractional part in the first Brillouin zone.

(5) Using the DFT phonons interpolated on a fine grid over the first Brillouin zone, check whether for the given *h*, *k*, *l* there is a matching phonon frequency corresponding to ω within a given tolerance in meV, and compute the TDS intensity using equations (6) or (7) as appropriate and equation (8). Add this to the reciprocal-space voxel using the master map.

(6) Repeat until all wavelengths are covered.

This recipe has been coded in *OpenCL* to enable parallel computation using multicore CPUs and GPUs.

## 4. Results   

**Figure 3:**
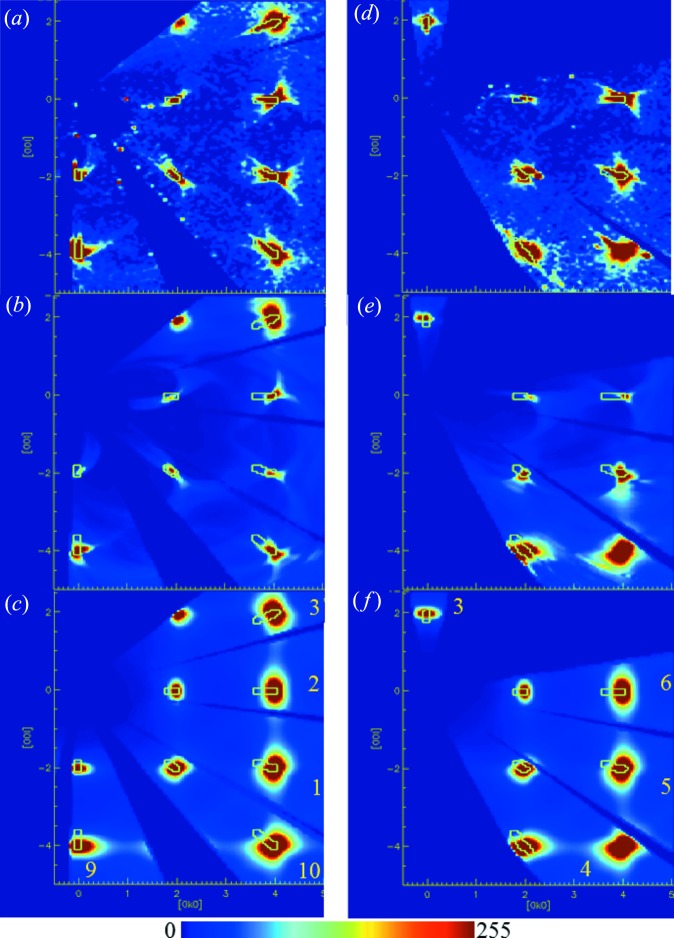
Comparison of the (0*k* *l*) layer between experiment and theory. (*a*) Experimental data from the synthetic NaCl crystal at room temperature recorded in one orientation. Contour lines are guides for the eye for the shape of the Bragg peaks at the base of the peaks. (*b*) Computation of phonon excitations and (*c*) thermal diffuse scattering in the quasi-static approximation. Views (*d*), (*e*), (*f*) correspond to (*a*), (*b*), (*c*) for the same NaCl specimen in a different orientation as described in the text. The numbers in (*c*) and (*f*) refer to the detector bank contributing to this section and correspond to the labelling in Fig. 2 ▸.

Sections through the (0*k* *l*) plane are shown in Fig. 3 ▸(*a*) employing data from the high-angle, 90° and low-angle banks. With reference to Fig. 2 ▸, which illustrates the detector numbering used in this paper, the high-angle banks are detectors 1, 6 and 10, the 90° banks are detectors 2, 5, 7, 9 and 11, and the low-angle banks are detectors 3, 4 and 8, respectively.

Diffuse scattering is concentrated close to the Bragg spots and takes the appearance of a butterfly shape. However, comparing the data from the various detectors, it becomes apparent that the diffuse scattering in the high-angle detectors near the Bragg spots is geometrically distorted rather than symmetric as would be expected. Weak arcs join or can be thought of as joining various neighbouring reflections on either side of the Bragg peak and these arcs are absent in the data from the low-angle banks. It should be noted that the indexing is taken as determined by the software. The assignment of which Miller index is *h*, *k* or *l* merely corresponds to a permutation of the symbols and is not relevant for comparing the various data sets. Sections were chosen to provide as large a coverage as possible. More complete volumetric data sets are available in the form of movies in the supplementary information.

NaCl phonons have been determined experimentally (Raunio *et al.*, 1969 ▸), albeit over a somewhat limited reciprocal space, and can quite easily be computed using state-of-the-art density functional codes such as *CASTEP* and *VASP*. NaCl is often included as an example tutorial for learning these programs, with the resulting phonon dispersion curves closely matching the experimental ones. Owing to the limited comparison and the fact that DFT phonons are calculated at 0 K with no adjustable parameters, only qualitative agreement would be expected in our calculations below. In the absence of direct phonon excitations in the quasi-static treatment, the thermal diffuse scattering can be calculated as the sum over all the phonon branches at a given *q* point using the formulas (5) and (9). It should be noted that in all calculations only the diffuse scattering is computed. Bragg peaks are not included, as is common practice when modelling diffuse scattering (Welberry, 2004 ▸).

The presence of direct phonon excitations in the data requires a different treatment. When creating the reciprocal-space maps, the underlying assumption is that the scattering events are purely elastic, *i.e.* no energy transfer occurs and the initial and final momenta of the scattered neutron have the same magnitude. Neither of these conditions is fulfilled when inelastic scattering takes place, which is governed by energy and momentum conservation.

The computations were carried out in the detector geometry of the instrument and then mapped to crystallographic reciprocal space as a volumetric data set as outlined in §3. Of the order of 5600 incident wavelengths were used from 560 meV down to 1 meV covering the time-of-flight range measured in the detector. The calculations were performed on Intel Xeon E5-2687 CPUs comprising effectively 32 cores. Despite parallelization of the codes, the calculations were rather formidable. Computing a volumetric data set of 300 × 300 × 300 points took 24 h per detector module and 5600 wavelengths. Three modules were computed in parallel and hence it would take of the order of four days to compute these volumetric data sets for all 11 detector modules and one orientation/temperature of the crystal. For comparison the TDS patterns took ∼100 s per detector module. At this point, the codes have not been fully optimized for speed or memory and further gains are possible. For each wavelength, the phonon spectrum was calculated for cases of both up and down scattering and all resulting maps were summed. Only inelastic events arising from exciting phonons from the ground state and *vice versa* were considered. Events such as energy transfer between occupied phonon states, multiphonon scattering and higher harmonics, *i.e.* any processes involving more than one phonon, were not included and did not appear essential as their contribution is thought to diminish rapidly. The resulting reciprocal-space maps were not convoluted with the instrument resolution. The time-of-flight peak shapes are typically asymmetric along the time-of-flight direction, leading to a tail at the base of the peak that points towards the origin of reciprocal space, giving the peaks a tear-drop shape in the reciprocal-space maps. The footprint of the Bragg peaks is indicated by contours in Figs. 3 ▸, 4 ▸ and 5 ▸ and also the supplementary movies. These were derived from the three-dimensional profile fitting routine in *SXD2001* after transforming them to reciprocal space and taking a 1% contour level from the maximum of each peak. The contours were elongated in the radial direction to better capture the asymmetric tail. Missing contours on some Bragg peaks are due to the peaks being too close to the detector edge and the integration not succeeding on them. However, they can be easily inferred from the neighbouring Bragg peaks. Trial runs were initially carried out to obtain a good value for the energy tolerance of 1 meV. It can be seen that the results obtained using both the standard TDS formalism and our method of explicitly taking into account direct phonon excitations resemble the data recorded with the low-angle bank, corresponding to detector 3 in Figs. 3 ▸(*a*)–3(*c*). However, for the 90° [detectors 2 and 9 in Figs. 3 ▸(*a*)–3(*c*)] and backscattering data (detectors 1 and 10) using the latter formalism results in a qualitatively better agreement compared with the TDS formalism with the observed diffuse scattering. A more complete illustration of the inelastic effects as they occur throughout reciprocal space and away from the (0*k* *l*) layer is contained in the movies in the supplementary information. It was already noted by Willis (1986 ▸) that inelastic effects would best be seen in backscattering.

**Figure 4:**
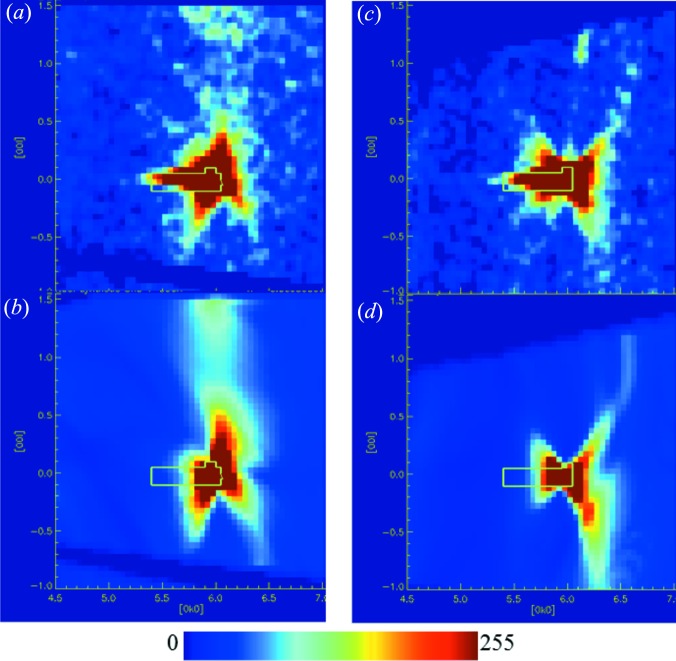
Comparison of an enlarged area around the 040 reflection between experiment and theory. (*a*) Experimental data from the synthetic NaCl crystal at room temperature recorded in one orientation, (*b*) computation of phonon excitations, (*c*) and (*d*) correspond to (*a*) and (*b*), respectively, for the rotated crystal. Contours are as in Fig. 3 ▸.

**Figure 5:**
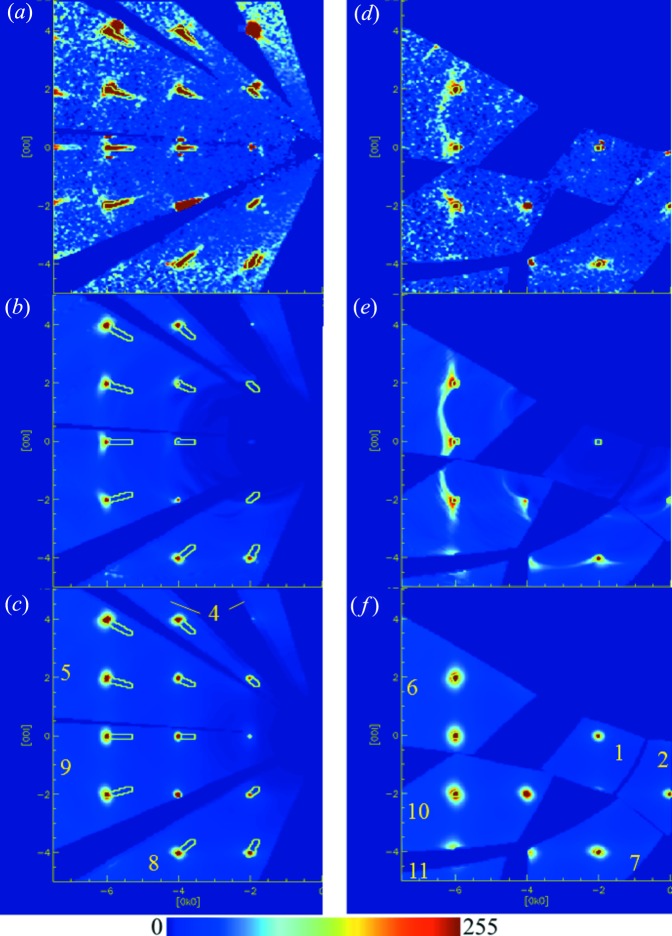
Comparison of the (0*k* *l*) layer between experiment and theory. (*a*) Experimental neutron data of the synthetic NaCl crystal at 20 K, (*b*) theoretical calculation using inelastic events at a temperature of 20 K, (*c*) thermal diffuse scattering in the quasi-static approximation. Views (*d*), (*e*), (*f*) correspond to (*a*), (*b*), (*c*), respectively, but are for the (4*k* *l*) layer.

To further ascertain the phonon origin of the scattering leading to the splitting of diffuse lines, two more experiments were conducted. In one, the same synthetic specimen was measured at room temperature in a different orientation by rotating the crystal around a vertical axis from ω = −150 to +150° and the diffuse scattering calculated accordingly using the full phonon dispersions and the quasi-static approach. The results are shown in Figs. 3 ▸(*d*)–3(*f*). An enlarged portion around the 040 reflection is shown in Fig. 4 ▸. Again, the diffuse scattering computed using the inelastic treatment shows better agreement. This illustrates that the diffuse features change appearance in the presence of inelastic effects as the crystal is rotated, whilst no changes are expected when using the quasi-static approximation. The former is expected, because upon rotating the crystal a given feature in reciprocal space is probed with neutrons of a different wavelength and hence energy. Intuitively, this can be understood from Bragg’s law: given a constant *d* spacing, when changing the scattering angle θ, the wavelength has to change. A similar effect, also reported by Welberry *et al.* (2003 ▸), is seen by comparing Figs. 3(*a*) and 5(*a*) in their paper. The angular dependence of this effect close to the Bragg peaks is discussed rather more formally by Schofield & Willis (1987) ▸. If the diffuse scattering changes appearance and diffuse lines appear split when measured in different orientations, this observation can be taken as an indication that these diffuse features originate from excitations arising in the sample rather than static lattice distortions. The second experiment again used the synthetic crystal in a somewhat different orientation compared with the room-temperature measurement, but this time the crystal was cooled to 20 K and the calculations carried out using this temperature and an energy tolerance of 1 meV. It is expected that this would change the population of the phonons leading to a more pronounced asymmetry in the intensity distribution of the features corresponding to the phonon excitation and annihilation. The results are shown in Fig. 5 ▸. The differences between the calculated patterns using the TDS and our approach in the (0*k* *l*) layer (Figs. 5 ▸ *a*–5*c*) are a bit more subtle and from a comparison with the experimental data it is not clear which would be preferred. In comparison, the (4*k* *l*) layer contains stronger diffuse features and again the inelastic treatment is clearly favoured. The intensity scales are the same as in Fig. 3 ▸. This allows assignment of the arc-like features on the higher- and lower-*Q* side of a Bragg peak to phonon emission and absorption, respectively. The second supplementary movie shows a side-by-side comparison of the 20 K data and calculations for a volumetric data set. Finally, we note that the inelastic effects break the symmetry of the diffraction pattern, and hence averaging of the diffuse scattering using the Laue symmetry of the crystal structure, as is commonly done in single-crystal diffuse scattering experiments, should be avoided in this case.

## 5. Summary and conclusions   

In this paper, we have illustrated the effect of inelastic excitations that can occur in a time-of-flight neutron single-crystal Laue experiment. The signal arising from one-phonon excitations takes the form of split arcs originating from Bragg reflections, and this is most pronounced in the data from the backscattering banks whilst being virtually absent in the low-angle detectors. The consequence is the breaking of the symmetry of the diffraction pattern, which means that such data should not be symmetrized using the Laue symmetry as is commonly done. Furthermore, recording of the diffuse scattering at a much lower temperature results in a pronounced asymmetry in the intensities of the diffuse split arcs on the high- and low-*Q* side of Bragg reflections due to the change in the phonon population. The 1/ω factor in equations (6) and (7) makes the acoustic modes the dominant contribution close to the Bragg reflections. Further away there is a dependence on the details of the phonon dispersions of a particular material, *e.g.* whether branches cross and the relative alignment of the eigenvectors and **Q** vector. Close to the *h*00, 0*k*0 and 00*l* reflections these would be the longitudinal acoustic modes allowing the derivation of the sound velocity as reported by Willis *et al.* (1986 ▸). The effects have been computed combining the TDS formulas for the inelastic scattering with phonon eigenvectors and frequencies as well as the temperature, and simulating the experiment in the actual instrument geometry using many wavelengths as a discretized representation of the incident white beam. It has been shown that in the case of neutron diffraction at a pulsed source the quasi-static approach is occasionally insufficient and the new approach presented in this paper provides a better way to simulate such features, despite the phonons being at the origin in both cases, *i.e.* the underlying physical cause of the diffuse scattering is the same. Whilst such effects may be considered a nuisance when modelling diffuse scattering using a Monte Carlo approach (Welberry, 2004 ▸), they can help in augmenting the information and classifying diffuse features with respect to their dynamic or static origin (Welberry *et al.*, 2003 ▸). If *ab* *initio* phonons, or magnons in the case of magnetic diffuse scattering, are available these can be used to aid in the interpretation of such features and contrasting them with diffuse scattering arising from other types of disorder. In the case of magnons, polarized neutrons can further establish their magnetic origin (Brückel & Schweika, 2002 ▸). As more instruments similar to SXD are now operational at other spallation neutron sources such as TOPAZ at the Spallation Neutron Source (Oak Ridge National Laboratory, USA) or SENJU at J-PARC (Ibaraki Prefecture, Japan), it is anticipated that such inelastic features will increasingly be observed (Schultz *et al.*, 2014 ▸; Tamura *et al.*, 2012 ▸). It can also be used complementarily to neutron powder inelastic experiments or optical spectroscopies such as IR, UV–Vis and Raman. The work presented in this paper can serve as a starting point to address a number of interesting topics arising from such effects, such as to what extent one can derive phonon dispersion curves, possibly taking into account several crystal orientations. This does not appear straightforward, as there is no simple geometric mapping between features before and after rotating the crystal. The conditions for phonon excitations are different, and the patterns are a superposition of phonon excitation curves measured with many different wavelengths and, in addition, are dependent on the instrument geometry. Another point concerns the TDS correction on Bragg intensities: Schofield & Willis (1987 ▸) noted that in the presence of inelastic excitation effects under favourable conditions phonon excitations may be forbidden around certain reflections, leading to Bragg intensities completely free of TDS, but details of the influence in the presence of such effects in general have not been worked out to date. Finally, as further extensions of this work one may envisage using such effects to fit either model potentials (Holt * et al.*, 1999 ▸) or coupling this with DFT to derive better model potentials, possibly including other kinds of spectroscopic data as well. From a theory point of view, DFT has the advantage of being applicable to different kinds of materials by choice of the appropriate functional without the need to fit many parameters to a model potential. The computational effort in the current work for our method and the DFT calculations on top of this seem rather discouraging for the time being but this may change sometime in the future with growing computing power.

## Supplementary Material

Quicktime movie scanning layer-by-layer through reciprocal space comparing the 20 K data measured on the SXD diffractometer on the left with the corresponding computed section using the inelastic excitation formalism on the right.. DOI: 10.1107/S1600576715010912/ks5457sup2.mov

Quicktime movie scanning layer-by-layer through reciprocal space comparing the room-temperature data measured on the SXD diffractometer on the left with the corresponding computed section using the inelastic excitation formalism on the right.. DOI: 10.1107/S1600576715010912/ks5457sup1.mov

## Figures and Tables

**Table 1:** List of angular ranges () for the various detector modules of the SXD instrument

Detector module	(centre range)	(centre range)
1	142.5 23	0 23
2	90 23	0 23
3	37.5 23	0 23
4	37.5 23	0 23
5	90 23	0 23
6	142.5 23	0 23
7	90 37	45 20
8	0 37	45 20
9	90 37	45 20
10	180 37	45 20
11	0 180	90 25
